# Long-Term Real-World Effectiveness and Safety of Recombinant ADAMTS13 in Congenital Thrombotic Thrombocytopenic Purpura: A Multicenter Retrospective Follow-Up Study in Poland

**DOI:** 10.7759/cureus.119552

**Published:** 2026-11-12

**Authors:** Aleksandra Laguna, Maria Szczepanska, Danuta Pietrys, Magdalena Wojdalska, Elzbieta Trembecka-Dubel, Milosz Jazdon, Walentyna Balwierz, Pawel Laguna

**Affiliations:** 1 Sferocyt Scientific Society at the Department of Paediatric Oncology, Haematology and Transplantology, Medical University of Warsaw, Warsaw, POL; 2 Department of Paediatrics, Medical University of Silesia, Katowice, POL; 3 Department of Paediatric Oncology and Haematology, Jagiellonian University Medical College, Krakow, POL; 4 Department of Paediatrics, Oncology and Haematology, Medical University of Lodz, Lodz, POL; 5 Department of Paediatric Oncology, Haematology and Transplantology, Poznan University of Medical Sciences, Poznan, POL; 6 Department of Paediatric Oncology, Haematology and Transplantology, Medical University of Warsaw, Warsaw, POL

**Keywords:** congenital thrombotic thrombocytopenic purpura, enzyme replacement therapy, long-term follow-up, paediatric haematology, paediatrics, recombinant adamts13, thrombotic microangiopathy (tma)

## Abstract

Background

Congenital thrombotic thrombocytopenic purpura (cTTP) is an ultrarare, inherited thrombotic microangiopathy caused by severe ADAMTS13 deficiency. Recombinant ADAMTS13 (rADAMTS13) provides targeted enzyme replacement, offering an alternative to plasma-based prophylaxis. Although clinical trials have demonstrated encouraging outcomes, evidence regarding long-term effectiveness and safety in routine clinical practice remains limited. This study evaluated extended hematological outcomes, acute thrombotic thrombocytopenic purpura (TTP) events, treatment continuity, and safety during rADAMTS13 prophylaxis.

Methods

This multicenter retrospective observational study included 10 patients with cTTP treated at 5 specialist centers in Poland. Nine patients belonged to a previously published cohort, and one additional patient was included. All had previously received fresh frozen plasma (FFP) and subsequently received intravenous rADAMTS13 at 40 IU/kg every two weeks. Clinical records and standardized physician questionnaires were reviewed through May 2026. Laboratory parameters were evaluated at pretreatment baseline, an initial on-treatment assessment, and extended follow-up. Follow-up lasted approximately 2 to 4 years in the original cohort and 11 months in the additional patient.

Results

All 10 patients continued regular rADAMTS13 prophylaxis without permanent treatment discontinuation or regimen modification. Mean platelet counts were 201.3 ± 93.2 × 10⁹/L at baseline, 252.0 ± 50.5 × 10⁹/L initially, and 193.1 ± 35.6 × 10⁹/L at extended follow-up. All patients had platelet counts within the reference interval at the latest assessment, compared with seven at baseline. Mean lactate dehydrogenase decreased from 239.7 ± 78.1 to 183.8 ± 25.7 U/L. Two patients (20%) experienced clinically diagnosed acute TTP episodes associated with intercurrent infections and required supplementary treatment. Incomplete event-specific laboratory documentation prevented independent verification against standardized criteria. No new strokes or clinically documented renal deterioration were reported. No ADAMTS13 inhibitors were detected in May 2026, and no serious adverse events attributed to rADAMTS13 were documented.

Conclusions

Extended rADAMTS13 prophylaxis was associated with favorable hematological findings at the available assessments, sustained treatment continuity, and an encouraging safety and tolerability profile. These observations provide reassuring real-world evidence supporting its potential value in the long-term management of cTTP. Nevertheless, infection-associated acute TTP events demonstrate the importance of continued clinical surveillance and timely access to additional treatment. Larger prospective studies are needed to confirm long-term effectiveness and safety.

## Introduction

Congenital thrombotic thrombocytopenic purpura (cTTP), also known as Upshaw-Schulman syndrome, is an ultrarare inherited thrombotic microangiopathy caused by severe deficiency of ADAMTS13, a metalloprotease responsible for cleaving ultralarge von Willebrand factor multimers. Deficient ADAMTS13 activity permits the accumulation of these multimers, promoting platelet aggregation, microvascular thrombosis, and the development of thrombocytopenia and microangiopathic hemolytic anemia. Clinical manifestations are heterogeneous, ranging from presentations in early infancy to disease onset in adulthood, and may include potentially life-threatening neurological, renal, and other organ complications. The International Hereditary Thrombotic Thrombocytopenic Purpura Registry highlighted the considerable variability in disease presentation and clinical course [1]. More recently, longitudinal data from the United Kingdom TTP Registry demonstrated the substantial burden of long-term complications, reinforcing the importance of effective prophylaxis and continued clinical monitoring [2].

Historically, the management of cTTP has relied on plasma-based therapies, particularly fresh frozen plasma (FFP), to replenish deficient ADAMTS13 activity. Although regular plasma prophylaxis can reduce acute disease episodes and the risk of organ damage, its long-term use is associated with important limitations, including hypersensitivity reactions, volume-related complications, and the practical burden of repeated infusions [2,3]. The development of recombinant ADAMTS13 (rADAMTS13) has represented a significant therapeutic advancement by enabling targeted replacement of the deficient enzyme without reliance on donor plasma. In a randomized phase III trial, Scully et al. demonstrated favorable efficacy and safety outcomes with rADAMTS13 compared with plasma-based therapy in the interim analysis [3]. The subsequent final analysis of the same trial, published in 2026, provided further evidence supporting the clinical benefits and favorable safety profile of recombinant enzyme replacement [4]. Reflecting these advances, the 2025 focused update of the International Society on Thrombosis and Haemostasis (ISTH) guidelines issued a strong recommendation favoring rADAMTS13 over plasma infusion for prophylactic treatment of patients with cTTP in remission [5].

Despite these developments, evidence regarding the long-term effectiveness and safety of rADAMTS13 in routine clinical practice remains limited, particularly among patients receiving prophylaxis from childhood and continuing treatment into adulthood. Real-world longitudinal observations are therefore important for evaluating hematological outcomes, clinical events arising during intercurrent illnesses, and the practical challenges associated with prolonged treatment. In Poland, Laguna et al. reported the initial real-world experience of pediatric patients receiving rADAMTS13 across five specialist centers, demonstrating encouraging hematological responses and treatment tolerability [6]. Windyga et al. also reported Polish real-world outcomes with rADAMTS13 prophylaxis in a letter to the editor, describing favorable clinical and hematological findings following the transition from plasma-based treatment to recombinant enzyme replacement [7]. Nevertheless, extended longitudinal follow-up remains valuable for characterizing treatment continuity, subsequent clinical events, and safety beyond the initial treatment period.

Building upon our previously published pediatric cohort, the present multicenter retrospective study extends clinical and laboratory follow-up through May 2026 and incorporates one additional patient with cTTP. The study aims to evaluate the long-term real-world effectiveness and safety of prophylactic rADAMTS13 by examining longitudinal hematological and biochemical parameters, infection-associated clinical events, requirements for supplementary treatment, treatment continuity, and adverse events. By providing extended observations from an established multicenter cohort, this investigation seeks to contribute further evidence regarding the long-term management of patients with this rare and potentially life-threatening disorder.

## Materials and methods

This multicenter, retrospective observational follow-up study evaluated the long-term effectiveness and safety of rADAMTS13 prophylaxis in patients with cTTP in Poland. The study extends a previously published investigation by Laguna et al., which reported the initial real-world experience of nine pediatric patients receiving rADAMTS13 [6].

The study was conducted at the same five Polish centres throughout the observation period, comprising four pediatric hematology units and one pediatric nephrology unit, located in Warsaw, Kraków, Poznań, Lodz, and Zabrze. The extended cohort included the original nine patients (P1-P9) and one additional patient (P10), who initiated treatment on June 1, 2025, and was not included in the previous publication. Patients who reached adulthood remained under the care of their original pediatric centres, allowing continued clinical follow-up without a change in participating centers.

The observation period extended from the first initiation of rADAMTS13 treatment in August 2022 through the latest available assessments up to May 2026. The original nine patients (P1-P9) initiated treatment during 2022-2023 and had approximately two to four years of follow-up by May 2026. In contrast, P10 initiated treatment on June 1, 2025, and had approximately 11 months of follow-up.

Study population and diagnostic criteria

The study included 10 patients with cTTP, comprising 5 females (50%) and 5 males (50%). In the original nine patients, cTTP had been diagnosed before treatment initiation based on severe ADAMTS13 deficiency, with activity below 2% and no detectable neutralizing ADAMTS13 inhibitors at diagnosis, as previously reported [6]. The additional patient fulfilled the same diagnostic criteria.

Historical clinical data included initial disease manifestations, episodes of thrombocytopenia or anemia, neurological complications, deterioration in renal function, and previous treatment with FFP.

All patients with confirmed cTTP receiving prophylactic rADAMTS13 at the five participating centers during the study period were included. There was no untreated control group. Given the rarity of cTTP and the limited number of available patients, no formal sample size calculation was performed.

Treatment protocol and administration settings

All patients received intravenous prophylactic rADAMTS13 at a dose of 40 IU/kg body weight every two weeks. The regular prophylactic regimen was maintained throughout follow-up without permanent treatment discontinuation or changes in the established dose or prophylactic dosing interval.

Treatment was initially provided under expanded-use authorization granted by the Polish Ministry of Health. Before January 2026, rADAMTS13 was administered in different settings according to individual arrangements. Two patients received treatment at home, one at a primary healthcare facility, and seven in hospitals.

In January 2026, implementation of the Polish national drug program B.164 initiated a transition toward hospital-based rADAMTS13 administration at the participating centers [8]. Previously established medication storage and distribution arrangements temporarily remained in place during the transition. Following implementation of the new system, scheduled prophylactic infusions were administered exclusively in hospital settings.

Any additional rADAMTS13 doses or FFP administered during intercurrent clinical events were recorded separately from regular prophylactic treatment.

Data collection and laboratory assessment

Clinical and laboratory information was collected retrospectively from patient medical records at the five participating centers. Treating physicians completed an identical standardized data-collection questionnaire covering patient demographic characteristics, the latest available laboratory results, treatment modifications, FFP administration, neurological events, deterioration in renal function, recurrent thrombocytopenia or anemia, and treatment administration settings. The questionnaire is provided in the Appendices.

Additional safety information, including infusion-related reactions, hypersensitivity reactions, thrombotic events, bleeding complications, and other adverse events, was obtained from the available medical documentation rather than from separate questions within the standardized questionnaire.

For patients P1-P9, historical laboratory observations were obtained from the dataset used in the original study covering 2022-2024 [6]. These included pretreatment baseline measurements and early on-treatment assessments. The historical data had been reported previously and were reused in the present study to permit longitudinal comparison with the extended follow-up observations.

Patient P10, who began treatment in June 2025, was not part of the original published cohort. Her available pretreatment and early on-treatment measurements were subsequently added to the expanded study dataset and were distinguished from the previously published observations.

The extended laboratory follow-up consisted of the latest available measurements obtained through May 2026. No additional intermediate laboratory time points were included in the longitudinal comparisons.

The laboratory parameters evaluated were hemoglobin concentration (g/dL), platelet count (×10⁹/L), lactate dehydrogenase (LDH; U/L), reticulocyte count (%), total bilirubin, and serum creatinine. Routine follow-up blood samples were obtained before the next scheduled prophylactic rADAMTS13 infusion.

Laboratory testing was performed at the local laboratories routinely used by the participating centers. No central laboratory was employed. Historical total bilirubin and serum creatinine measurements recorded in µmol/L were converted to mg/dL before quantitative analysis.

Numerical reticulocyte measurements were included where available. When results were documented only as being within the reference range, they were retained for descriptive assessment rather than treated as numerical observations. ADAMTS13 inhibitor levels were assessed annually during routine clinical care and reported in Bethesda units per milliliter (BU/mL). 

Patients were identified in the analysis using coded study identifiers (P1-P10).

Study objectives and outcome measures

The primary objective was to evaluate the long-term real-world effectiveness of prophylactic rADAMTS13 in patients with cTTP by assessing clinical outcomes and changes in laboratory parameters over the extended observation period. The principal laboratory measures of effectiveness were platelet count and LDH concentration. Additional measures included hemoglobin concentration, reticulocyte count, total bilirubin, and serum creatinine.

Clinical outcomes included clinically diagnosed acute TTP episodes, other episodes of thrombocytopenia, recurrent anemia, transient ischemic attack or stroke, deterioration in renal function, unplanned hospital attendance or admission, and the need for supplementary rADAMTS13 or FFP treatment. Treatment continuity and changes in the location of treatment administration were also evaluated. Immunogenicity was assessed using the available ADAMTS13 inhibitor measurements.

Safety outcomes included infusion-related reactions, hypersensitivity reactions, thrombotic events, bleeding complications, and other reported adverse events, including serious events potentially associated with treatment. These outcomes were assessed retrospectively using the previously published historical data and additional clinical and laboratory observations available through May 2026.

Clinical event and safety assessment

Clinical events occurring during rADAMTS13 prophylaxis were identified through a retrospective review of medical records and the standardized questionnaires completed by the treating physicians. For each event, the available information included the associated clinical trigger, relevant laboratory abnormalities, additional therapeutic interventions, hospital attendance or admission, and subsequent clinical course. Particular attention was given to infection-associated episodes of thrombocytopenia and events requiring treatment beyond the established prophylactic regimen.

Acute TTP episodes were identified from the treating physicians’ clinical assessments and the available medical records. Associated clinical features, laboratory abnormalities, and additional treatment were documented when available. Where contemporaneous platelet counts or LDH measurements were missing, events were retained as clinically diagnosed acute TTP episodes but could not be independently assessed against standardized laboratory-based criteria.

Reported intercurrent illnesses and clinical complications were distinguished from adverse reactions attributed to rADAMTS13 whenever sufficient information was available.

Statistical analysis

Given the small sample size and retrospective observational design, the statistical analysis was primarily descriptive. Continuous variables were summarized using means and standard deviations (SD). Categorical variables were presented as absolute frequencies and percentages.

For patients P1-P9, laboratory parameters were assessed at three available observation points: pretreatment baseline, the initial on-treatment assessment reported in the previous study, and the latest available extended follow-up assessment through May 2026. Patient P10 was evaluated using her available pretreatment, early on-treatment, and final follow-up measurements. Her historical observations were considered separately from those of the original nine-patient cohort. 

Within-patient changes in platelet count, LDH, hemoglobin concentration, total bilirubin, and serum creatinine were evaluated descriptively using paired measurements where available. Comparisons involving the previously published cohort were restricted to P1-P9, while descriptive summaries of the extended cohort included all 10 patients with available data. 

Clinical events, additional treatment requirements, treatment continuity, and safety outcomes were summarized descriptively. Given the small sample size and the descriptive nature of the study, no formal inferential hypothesis testing was performed. Missing laboratory measurements were not imputed.

Ethical considerations

The original rADAMTS13 treatment was provided under authorization granted by the Polish Ministry of Health for the temporary supply of the medication. This authorization did not stipulate prior bioethics committee approval as a condition of treatment. The Ministry's authorization concerned access to rADAMTS13 and did not constitute ethics committee approval for the present research.

Following the introduction of the Polish national drug program B.164 in January 2026 [8], rADAMTS13 treatment continued as part of routine clinical care. The additional patient (P10), who initiated treatment on June 1, 2025, was subsequently enrolled in program B.164. Appropriate informed consent for treatment was obtained. Participation in the national therapeutic program did not itself require separate bioethics committee approval.

The present investigation was a multicenter, non-interventional, retrospective observational analysis of existing clinical and laboratory data collected during routine patient care at five specialist centers in Poland, coordinated by the Medical University of Warsaw. No additional therapeutic interventions or research-specific procedures were undertaken.

Under Articles 21 and 29 of the Polish Act of 5 December 1996 on the Professions of Physician and Dentist (Ustawa o zawodach lekarza i lekarza dentysty) [9], the statutory requirement for bioethics committee approval applies to medical experiments. As the present investigation involved only the retrospective analysis of existing routine-care clinical data, without experimental interventions or research-specific procedures, it did not constitute a medical experiment within the meaning of Article 21 and was therefore considered outside the scope of the ethics approval requirement for medical experiments under Article 29.

The applicability of this regulatory framework and the relevant institutional requirements was verified at all five participating centers in Warsaw, Kraków, Poznań, Łódź, and Zabrze. Based on the verified institutional requirements at all five participating centers, no additional bioethics committee review was required for this retrospective investigation, including the analysis of data relating to patient P10. No formal ethics committee approval or exemption certificate was issued for the study.

Written informed consent for treatment and open-access publication of de-identified clinical information was obtained from patients or their parents or legal guardians, as applicable, including P10. Patient information was reported using coded identifiers without direct personal identifiers. The study was conducted in accordance with the principles of the Declaration of Helsinki.

## Results

Patient characteristics and treatment continuation

The extended cohort comprised 10 patients with cTTP, including 5 females (50%) and 5 males (50%). Nine patients (P1-P9) had been included in the previous publication [6] and had approximately two to four years of follow-up by May 2026, having initiated rADAMTS13 prophylaxis during 2022-2023. One additional patient (P10), who initiated treatment on June 1, 2025, and had approximately 11 months of follow-up, was included in the extended analysis. Age at treatment initiation ranged from 2 to 17 years. All patients continued to be followed at the original five pediatric centers, including those who reached adulthood during observation. All 10 patients had received FFP in the past before initiating rADAMTS13 prophylaxis.

Based on the documented dates of treatment initiation and the study follow-up cutoff in May 2026, the estimated durations of rADAMTS13 prophylaxis were approximately 33 months for P1, 43 months for P2, 45 months for P3, 44 months for P4, 44 months for P5, 33 months for P6, 43 months for P7, 43 months for P8, and 11 months for P10. For P9, the exact date of the first rADAMTS13 administration was unavailable; therefore, the individual follow-up duration could not be reliably calculated.

All 10 patients remained on intravenous rADAMTS13 prophylaxis at 40 IU/kg every 2 weeks through the latest available follow-up assessments up to May 2026. No permanent treatment discontinuations or changes to the scheduled prophylactic regimen were documented. Before January 2026, rADAMTS13 was administered at home in two patients (20%), at a primary healthcare facility in one patient (10%), and in hospital in seven patients (70%). Following implementation of the Polish national drug program B.164 and its transitional arrangements, regular prophylactic infusions were administered in hospital settings.

Longitudinal laboratory outcomes

For each patient, pretreatment and initial on-treatment laboratory measurements were compared with the latest available follow-up results through May 2026. Across all 10 patients, mean platelet count was 201.3 ± 93.2 × 10⁹/L before treatment, 252.0 ± 50.5 × 10⁹/L at the initial on-treatment assessment, and 193.1 ± 35.6 × 10⁹/L at extended follow-up. Three patients had platelet counts below 150 × 10⁹/L at baseline; no patient had a platelet count below this threshold at either subsequent assessment. At extended follow-up, platelet counts ranged from 152 to 272 × 10⁹/L, within the conventional reference interval of 150-450 × 10⁹/L for all 10 patients.

Mean lactate dehydrogenase (LDH) concentration was 239.7 ± 78.1 U/L at baseline, 199.0 ± 35.1 U/L initially, and 183.8 ± 25.7 U/L at extended follow-up. The mean paired baseline-to-final change was −55.9 U/L. Corresponding mean hemoglobin concentrations were 12.93 ± 1.38, 12.76 ± 1.09, and 13.48 ± 1.54 g/dL, respectively.

After harmonization of historical units, mean total bilirubin was 0.70 ± 0.43 mg/dL at baseline (n = 9), 0.52 ± 0.22 mg/dL initially (n = 9), and 0.55 ± 0.15 mg/dL at extended follow-up (n = 10). Mean serum creatinine was 0.51 ± 0.13 mg/dL at baseline (n = 9), 0.55 ± 0.13 mg/dL initially (n = 10), and 0.61 ± 0.15 mg/dL at extended follow-up (n = 10). Numerical reticulocyte data were incomplete at the final assessment; available clinical reports described these results as within the reference range. Table 1 summarizes the laboratory findings.

**Table 1:** Laboratory findings at the three available assessments Values are mean ± sample standard deviation. Historical observations for P1-P9 were reported in the previous study [6], whereas the historical observations for P10 were collected subsequently. Historical bilirubin for P8 and baseline creatinine for P3 were unavailable. Historical bilirubin for P6 and P7 and creatinine for P6 were converted from µmol/L to mg/dL before calculation. No missing results were imputed.

Parameter	Pretreatment baseline	Initial on-treatment	Extended follow-up
Hemoglobin (g/dL)	12.93 ± 1.38 (n=10)	12.76 ± 1.09 (n=10)	13.48 ± 1.54 (n=10)
Platelet count (×10⁹/L)	201.3 ± 93.2 (n=10)	252.0 ± 50.5 (n=10)	193.1 ± 35.6 (n=10)
LDH (U/L)	239.7 ± 78.1 (n=10)	199.0 ± 35.1 (n=10)	183.8 ± 25.7 (n=10)
Total bilirubin (mg/dL)	0.70 ± 0.43 (n=9)	0.52 ± 0.22 (n=9)	0.55 ± 0.15 (n=10)
Creatinine (mg/dL)	0.51 ± 0.13 (n=9)	0.55 ± 0.13 (n=10)	0.61 ± 0.15 (n=10)

Individual longitudinal responses

At extended follow-up, platelet counts were higher than pretreatment baseline in five patients and lower in five. Relative to the initial on-treatment assessment, platelet counts were lower in eight patients and higher in two, although all final values remained within the conventional reference interval. Final LDH concentrations were lower than baseline in seven patients and lower than the initial on-treatment assessment in seven patients. Hemoglobin was higher at final follow-up than at the initial assessment in eight patients.

In the original 9-patient cohort, mean platelet counts were 211.4, 251.1, and 197.0 × 10⁹/L at baseline, initial assessment, and extended follow-up, respectively. Corresponding LDH concentrations were 240.8, 198.3, and 183.7 U/L. For the additional patient P10, platelet counts were 110, 260, and 158 × 10⁹/L, and LDH concentrations were 230, 205, and 185 U/L at the three assessments.

Table 2 provides the paired patient-level platelet and LDH data. The corresponding changes are visualized in Figure 1 (panels A and B).

**Table 2:** Individual patient platelet counts and LDH concentrations Platelet count (PLT), × 10⁹/L; lactate dehydrogenase (LDH), U/L. All three observations are directly matched by patient. The pretreatment baseline and initial on-treatment values for patients P1–P9 were previously reported [6]. The corresponding values for P10 and the extended follow-up measurements for all patients are reported in the present study. The initial assessment and final follow-up were not separated by a common fixed interval across patients.

Patient	PLT baseline	PLT initial	PLT follow-up	LDH baseline	LDH initial	LDH follow-up
P1	299	291	192	171	155	174
P2	42	302	217	418	209	228
P3	187	193	272	185	159	205
P4	303	346	186	151	163	152
P5	284	199	152	323	243	160
P6	273	217	173	231	215	184
P7	210	243	211	236	260	210
P8	214	204	205	231	197	190
P9	91	265	165	221	184	150
P10	110	260	158	230	205	185

**Figure 1:**
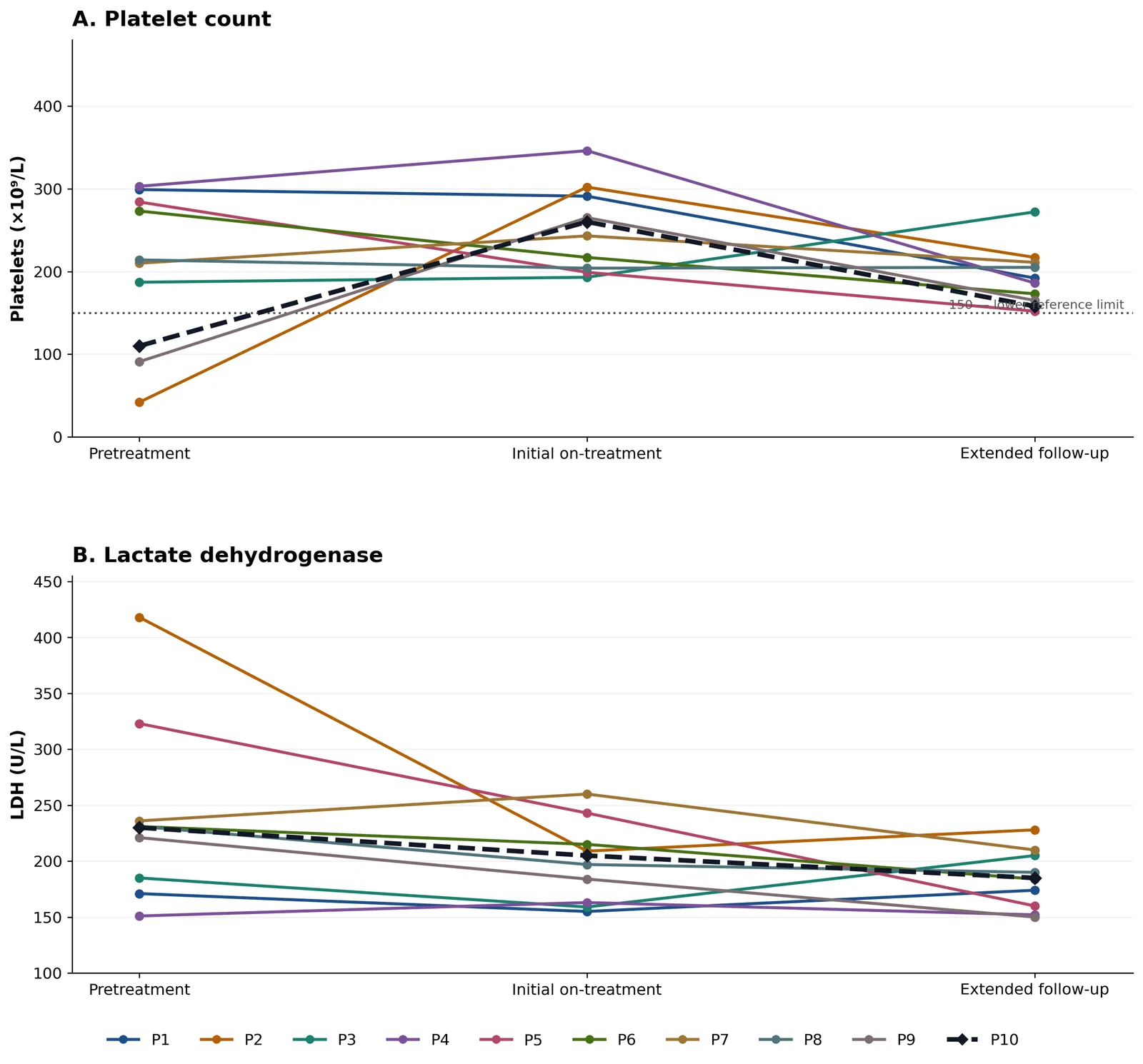
Individual longitudinal changes in (A) platelet counts and (B) LDH concentrations Each line represents one patient; P10 is distinguished by a dashed line. Horizontal spacing reflects three distinct observation points (pretreatment baseline, initial on-treatment assessment, and extended follow-up), rather than elapsed time. The pretreatment baseline and initial on-treatment values for patients P1–P9 were previously reported [6]. The corresponding values for P10 and the extended follow-up measurements for all patients are reported in the present study. The dotted horizontal line in panel A indicates a platelet count of 150 × 10⁹/L. LDH, lactate dehydrogenase

Infection-associated acute TTP events and additional treatment

Two patients (20%) experienced clinically diagnosed acute thrombotic thrombocytopenic purpura (TTP) episodes associated with intercurrent infections and required treatment in addition to scheduled prophylaxis. No additional rescue treatment was reported in the remaining eight patients.

In January 2026, P2 experienced an acute TTP episode associated with influenza A and clinically documented marked thrombocytopenia shortly before a scheduled rADAMTS13 infusion. The patient attended a regional hospital at the weekend when rADAMTS13 was not immediately accessible because the medication remained stored at a primary healthcare facility during the transition to the national drug program. FFP was administered, and the patient was discharged on the same day. An additional dose of rADAMTS13 was subsequently administered, and regular prophylaxis continued. Clinical recovery and subsequent normalization of platelet counts were reported.

In October 2025, P10 experienced an acute TTP episode associated with gastroenteritis, requiring hospitalization for two days. Two supplementary rADAMTS13 doses (40 IU/kg each) were administered 24 hours apart. According to the treating physician, the platelet count returned to the normal range within two days. Numerical platelet and LDH results obtained during the episode were unavailable for retrospective review. FFP was not administered, and regular prophylaxis was maintained without permanent modification.

Both events were diagnosed clinically as acute TTP episodes by the treating physicians. Because complete contemporaneous platelet and LDH measurements were unavailable for retrospective review, their classification could not be independently verified against standardized laboratory-based criteria.

Neurological and renal outcomes

No transient ischemic attacks, strokes, or new clinically documented deterioration in renal function were reported during the extended follow-up. No recurrent anemia requiring a change in scheduled prophylaxis was documented.

Safety and immunogenicity

Apart from the two clinically diagnosed acute TTP episodes described above, no additional thrombotic events, bleeding complications, infusion-related reactions, hypersensitivity reactions, or serious adverse events attributed to rADAMTS13 were documented in the reviewed follow-up records. The acute TTP episodes associated with influenza A in P2 and gastroenteritis in P10 were considered manifestations of the underlying disease rather than adverse reactions attributed to rADAMTS13.

Annual ADAMTS13 inhibitor surveillance was part of routine care. No ADAMTS13 inhibitors were detected at the May 2026 follow-up assessment, and no inhibitor-related modifications of the treatment regimen were reported.

## Discussion

This multicenter retrospective follow-up study provides additional real-world evidence regarding the long-term use of rADAMTS13 prophylaxis in patients with cTTP. By extending our previously published pediatric cohort [6] through May 2026 and incorporating one additional patient, the present study provides longer-term observations of hematological outcomes, treatment continuity, infection-associated clinical events, and safety under routine clinical conditions.

The principal findings were the maintenance of platelet counts within the conventional reference interval at the latest follow-up assessment in all 10 patients, lower mean LDH concentrations compared with pretreatment measurements, and continued administration of the established prophylactic regimen without permanent treatment discontinuation. Two patients experienced clinically diagnosed acute TTP episodes in association with intercurrent infections and required additional treatment; both subsequently continued their regular prophylactic schedules. These findings are consistent with hematological control at the available assessment points, while demonstrating that acute TTP episodes may occur despite ongoing prophylaxis.

Platelet count and LDH concentration are important markers of disease activity in cTTP, reflecting platelet consumption and microangiopathic processes associated with severe ADAMTS13 deficiency [3]. In the present cohort, mean platelet count increased after treatment initiation but was lower at the extended follow-up assessment than during the initial on-treatment evaluation. Nevertheless, all patients had platelet counts within the conventional reference interval at their latest assessment. This distinction is clinically relevant, as a decrease in platelet count between two measurements does not necessarily indicate loss of treatment effectiveness when the subsequent value remains within the reference range. However, the limited number of available laboratory assessments prevents conclusions regarding continuous hematological stability between visits.

Mean LDH concentration declined from 239.7 U/L before treatment to 183.8 U/L at extended follow-up, with seven patients demonstrating lower concentrations than at baseline. This pattern is compatible with reduced biochemical evidence of ongoing microangiopathic activity during rADAMTS13 prophylaxis. Hemoglobin concentrations were also maintained over the extended observation period. Mean total bilirubin concentrations were lower at extended follow-up than at pretreatment baseline, consistent with the overall pattern of favourable biochemical findings.

These observations are broadly consistent with previously published clinical trial evidence. In the phase III randomized crossover trial reported by Scully et al., prophylaxis with rADAMTS13 was associated with fewer manifestations of cTTP than plasma-based treatment, with no acute TTP episodes observed during rADAMTS13 prophylaxis in the interim analysis [3]. The final analysis, published in 2026, further supported the clinical benefit of recombinant enzyme replacement, reporting fewer subacute TTP events and thrombocytopenic manifestations during rADAMTS13 prophylaxis than during plasma-based treatment [4].

All 10 patients in the present cohort had previously received FFP. In the original nine-patient report, seven patients (77.8%) experienced complications related to FFP, predominantly allergic reactions, including one case of transfusion-related acute lung injury; six (66.7%) received premedication, including antihistamines with or without other agents [6]. In contrast, no serious adverse events attributed to rADAMTS13 were documented during the extended follow-up. Hematological and biochemical findings at the available assessments did not indicate consistent deterioration after the transition from plasma-based prophylaxis to rADAMTS13. These findings support continued disease control.

Experience in the United Kingdom provides a useful international comparison. In the UK TTP Registry, regular plasma-derived prophylaxis was associated with fewer acute TTP episodes and reduced organ complications, but symptoms and plasma-related treatment burdens persisted. Among patients receiving regular prophylaxis, 43 (58.9%) had transitioned to rADAMTS13, most commonly because of inadequate symptom control (53.5%), plasma reactions (30.2%), or subclinical disease activity (16.3%) [2]. These findings support the importance of evaluating treatment tolerability and patient burden alongside hematological control.

Additional real-world evidence has been reported by Windyga et al. in a letter to the editor, which described favorable clinical and hematological outcomes following the transition from FFP to rADAMTS13 prophylaxis [7]. While their report focused primarily on outcomes associated with treatment transition, the present investigation provides extended longitudinal follow-up of an established pediatric cohort, with particular emphasis on hematological outcomes, infection-associated clinical events, treatment continuity, and safety through May 2026.

The two clinically diagnosed acute TTP episodes associated with intercurrent infections highlight the importance of maintaining clinical vigilance during regular enzyme replacement therapy. Both patients required additional treatment despite their ongoing prophylaxis. Although the treating physicians identified these as acute TTP events, incomplete contemporaneous platelet and LDH measurements prevented independent verification against standardized laboratory-based criteria. Consequently, these clinically diagnosed events should not be presented as laboratory-confirmed episodes or compared directly with event rates from controlled trials using prespecified biochemical definitions.

The influenza-associated event also illustrates an organizational consideration in long-term treatment. Although the transition to Poland's national drug program B.164 established hospital-based administration of rADAMTS13, previously established storage arrangements remained temporarily in place during implementation. The resulting delay in access to treatment demonstrates the importance of clear arrangements for urgent medication availability, particularly outside routine healthcare operating hours. This observation concerns the organization of clinical care rather than a demonstrated failure of the prophylactic treatment itself. According to the European Medicines Agency product information, unopened lyophilized ADZYNMA can be stored at room temperature (up to 30 °C) for up to six months, provided that the expiry date is not exceeded; after room-temperature storage, it should not be returned to refrigeration. Home administration may be considered after appropriate clinical assessment and patient or caregiver training [10]. A future option for suitable patients could be access to one emergency dose at home to reduce delays during intercurrent illness. Such an arrangement would require appropriate prescribing, supervision, storage safeguards, and compatibility with Polish drug-program rules, and is not part of the current hospital-based arrangement.

The extended follow-up also provides additional information regarding treatment tolerability. Apart from the two clinically diagnosed acute TTP episodes, no additional thrombotic events, bleeding complications, infusion-related reactions, hypersensitivity reactions, or serious adverse events attributed to rADAMTS13 were identified in the reviewed clinical documentation. No ADAMTS13 inhibitors were detected at the May 2026 follow-up assessment, and no inhibitor-related treatment modifications were reported. These observations are broadly consistent with the favorable treatment-related safety findings of the phase III clinical trial and its final analysis [3,4]. Taken together, the absence of documented serious treatment-related adverse events and the negative inhibitor assessment in May 2026 are encouraging, while the small cohort cannot exclude uncommon complications.

Nevertheless, the absence of documented treatment-related adverse events in a cohort of 10 patients cannot exclude uncommon or delayed complications. In September 2026, the US Food and Drug Administration introduced a boxed warning for rADAMTS13 (Adzynma) following reports of neutralizing ADAMTS13 antibodies associated with serious outcomes [11]. Although no ADAMTS13 inhibitors were detected at the May 2026 follow-up assessment, immunogenicity observations were derived from routine clinical monitoring rather than a standardized prospective protocol. These considerations highlight the importance of continued safety surveillance and long-term immunogenicity monitoring.

The transition to hospital-based administration also raises questions regarding the practical burden of long-term treatment. Earlier arrangements included administration at home and in primary healthcare facilities, whereas the national drug program introduced hospital-based treatment for all patients. Although the change did not affect the prescribed prophylactic regimen, its implications for treatment convenience, quality of life, and healthcare resource utilization were not formally evaluated. These outcomes would be valuable areas for future investigation.

The principal strength of this study is the extended observation of an established cohort with an ultrarare inherited disease, using patient-level clinical and laboratory information collected from the same five participating centers. Inclusion of an additional patient and continued follow-up of individuals who reached adulthood provide information regarding the feasibility of maintaining prophylactic treatment over different stages of life. Furthermore, the use of standardized physician questionnaires supported consistency in clinical data collection.

Several limitations must be acknowledged. First, the retrospective design and small sample size limit the generalizability of the findings and prevent robust assessment of uncommon outcomes. Second, follow-up duration differed between patients, with P10 having a substantially shorter observation period than the original nine patients. 

Additional limitations include the use of local rather than centralized laboratories and incomplete laboratory documentation during clinically diagnosed acute TTP episodes. Clinical adverse event ascertainment was dependent on the completeness of retrospective medical records. Furthermore, the absence of formal quality-of-life assessments limited the evaluation of broader patient-centered outcomes.

## Conclusions

This multicenter retrospective study provides encouraging real-world evidence regarding the long-term effectiveness and tolerability of recombinant ADAMTS13 prophylaxis in patients with congenital thrombotic thrombocytopenic purpura. The findings support its potential role in maintaining hematological disease control during prolonged treatment, while highlighting the importance of continued clinical surveillance and long-term safety monitoring. Particular attention should be given to the management of intercurrent infections, during which additional treatment may be necessary despite regular prophylaxis. Ensuring timely access to emergency therapy, including outside routine healthcare operating hours, represents an important consideration for optimizing patient care. Although these observations are reassuring, the small cohort size, retrospective design, and variable follow-up durations limit the strength of the conclusions. Larger prospective studies with standardized clinical and laboratory monitoring are warranted to further establish long-term effectiveness, characterize immunogenicity and rare adverse events, and evaluate treatment burden and patient-reported quality-of-life outcomes.

## Appendices

Standardized physician follow-up questionnaire

Recombinant ADAMTS13 (rADAMTS13) Treatment Follow-Up

1. Patient information

Patient initials: __________________

Date of birth: __________________

Sex: __________________

2. Most recent laboratory results

Hemoglobin (Hb): __________ g/dL

Reticulocyte count: __________ %

Platelet count (PLT): __________ × 10⁹/L

Lactate dehydrogenase (LDH): __________ U/L

Total bilirubin: __________ µmol/L

Serum creatinine: __________ µmol/L

3. Has the patient required any modification of their treatment regimen?

☐ Yes

☐ No

4. Has the patient received fresh frozen plasma (FFP)?

☐ Yes

☐ No

5. Has the patient experienced a transient ischemic attack (TIA) or stroke?

☐ Yes

☐ No

6. Has the patient experienced any deterioration in renal function?

☐ Yes

☐ No

7. Has the patient experienced recurrent episodes of thrombocytopenia or anemia?

☐ Yes

☐ No

Additional clinical details, including any supplementary treatment administered (if applicable):

8. Where is rADAMTS13 administered?

Please select the appropriate setting.

☐ Specialist hematology/nephrology center

☐ Regional hospital

☐ Patient's home
